# Cryopreservation of jaguar (*Panthera onca*) sperm cells using different cryoprotectants and different thawing temperatures

**DOI:** 10.1590/1984-3143-AR2023-0009

**Published:** 2023-04-03

**Authors:** Pedro Nacib Jorge-Neto, Thiago Cavalheri Luczinski, Gediendson Ribeiro de Araújo, Letícia Alecho Requena, Rogério Silva de Jesus, Larissa Schneider Brandão Souza, Ricardo Zanella, Eliane Vianna da Costa e Silva, Thyara de Deco-Souza, Cristiane Schilbach Pizzutto

**Affiliations:** 1 Instituto Reprocon, Campo Grande, MS, Brasil; 2 Faculdade de Medicina Veterinária e Zootecnia, Universidade de São Paulo, São Paulo, SP, Brasil; 3 No Extinction, Corumbá de Goiás, GO, Brasil; 4 Faculdade de Medicina Veterinária, Centro Universitário de Brasília, Brasília, DF, Brasil; 5 Biotério Central, Faculdade Federal de Mato Grosso do Sul, Campo Grande, MS, Brasil; 6 Faculdade de Medicina Veterinária e Zootecnia, Universidade Federal de Mato Grosso do Sul, Campo Grande, MS, Brasil; 7 Escola de Ciências Agrárias, Inovação e Negócios, Universidade de Passo Fundo, Passo Fundo, RS, Brasil

**Keywords:** biobank, feline, infrared thermography, penile spines, reproduction

## Abstract

The cryopreservation of jaguar semen must be improved to produce high-quality biobanking doses. Until now, the rare studies of semen freezing in the species have only evaluated glycerol, always with a significant reduction in sperm quality in thawed semen. The purpose of this study was to assess the efficacy of three cryoprotectants, dimethylsulfoxide (DMSO), glycerol (GLY), and methanol (MET), in the cryopreservation of jaguar semen in an LDL-based extender, as well as the effect of thawing temperature on dosage quality. Five mature males with a history of reproduction were used. On the males, an infrared thermal image (IRT) was captured, the spicules and testes were analyzed, and the CASA system was used to evaluate the quality of fresh and thawed sperm. The superficial IRT was 4.6 ± 1.2 °C cooler than the anal sphincter, and the semen measured between 27.3 and 28.7 °C shortly after exiting the urethra. The total motility of fresh sperm was 55.3 ± 22.6%, and progressive motility was 36.3 ± 18%. The total motility of thawed sperm was 5.28 ± 2.51%, 4.49 ± %2.49, and 0.51 ± 0.62% for DMSO, GLY, and MET, respectively. DMSO and GLY performed better than MET, and there was no difference in thawing temperature (37°C 30 s vs. 50°C 12 s). All animals exhibit a considerable level of morphological changes in sperm. Low amounts of total and progressive motility were found in the thawed sperm. Males with a high level of sperm morphological changes were found to be fertile, but the lone male with normospermia was infertile. Thus, we contest the applicability of the commonly used morphological classification for bovines to felid species.

## Introduction

It is well established that Assisted Reproduction Technologies (ART) are valuable tools for the genetic propagation of endangered species. To enable ART in jaguars (*Panthera onca*), researchers focused on improving sperm collection and cryopreservation from captive and wild individuals (Araujo et al., 2018; Morato et al., 2001; Silva et al., 2019a)​. During the last few years, pharmacological collection has gradually replaced electroejaculation (EE) as the preferred approach for semen collection in felids (Araujo et al., 2018; Lueders et al., 2012; Zambelli et al., 2008). This method offers the advantage of collecting more concentrated samples without subjecting the animal to electrical stimuli, which can affect the maintenance of anesthesia. Another benefit of the pharmaceutical collection is the near-total elimination of urine contamination, which is common with EE collections (Araujo et al., 2018; Lueders et al., 2012; Zambelli et al., 2008).

Besides the successes in semen collection, cryopreservation still needs refinement, aiming to improve the quality of thawed sperm. As in other felids, the Tris-Egg-yolk extender is the method of choice for semen cryopreservation in jaguars, using 4 to 7.5% glycerol as the cryoprotectant (Araujo et al., 2020a; Paz et al., 2000, 2007; Santos et al., 2022; Silva et al., 2020; Swanson et al., 1996). Although egg yolk is widely used, its instability and semen export restrictions necessitate the investigation of alternatives that have been used in other species, like soy lecithin and low-density lipoproteins (Bencharif et al., 2008; Lambo et al., 2012). When it comes to semen cryopreservation in free-living animals, egg yolk must be replaced, as it does not meet the logistical requirements of fieldwork. Also, no effort has been made to evaluate different cryoprotectants for the cryopreservation of jaguar semen. Cryoprotectant toxicity differs among species (Buranaamnuay, 2017; Deco-Souza et al., 2013), and therefore even if glycerol is the cryoprotectant of choice for the majority of feline species (Araújo et al., 2021a; Deco-Souza et al., 2013; Lorton, 2022), it is essential to evaluate if it is the best option for jaguars. For instance, among canids, maned wolves tolerate DMSO better, while red wolf responds better to glycerol (Franklin et al., 2018; Johnson et al., 2014).

The objective of the present study, therefore, was to compare the efficacy of three different cryoprotectants, dimethylsulfoxide (DMSO), glycerol (GLY), and methanol (MET) in the cryopreservation of jaguar semen in an LDL-based extender, as well as the effect of thawing temperature on dose quality.

## Methods

This study was conducted at NEX - No Extinction (Corumbá de Goiás, GO, Brazil; 15°51'32.3"S 48°28'32.1"W) facility on March 2022; it had authorization for scientific activities issued by SISBIO / ICMBio / MMA under no. 57293-12 and was approved by the Ethics Committee on Animal Use of the School of Veterinary Medicine and Animal Science of the University of São Paulo (CEUA/FMVZ) under nº. 1883210222. Under the protocol A9554A0, the genetic heritage accessions of both individuals were recorded in Brazilian National System for the Management of Genetic Heritage and Associated Traditional Knowledge (SISGEN).

### Animals and experimental design

Reproductively mature and healthy male jaguars (*n* = 5) were used, aged between 24 and 110 months (mo; Table 1) and weighing between 53 and 83 kg. One of the males was born in captivity, while the others are wild but were under human care for at least 24 months. Animals have *ad libitum* access to clean, fresh water, and their diet consists primarily of pork with bones and may include beef and chicken, as well as a commercial supplement (Aminomix Gold, Vetnil) for animal usage. They were housed with (Ferinha) or near females in outdoor enclosures that provide a natural setting (with substrate and vegetation), sun exposure, a swimming pool, a den, a lock-away area, and a security corridor. The animals also receive environmental enrichment to reduce the stress associated with captivity. They are naturally exposed to sunshine, temperature fluctuations, and precipitation, therefore maintaining a natural circadian rhythm. Feeding the animals and maintaining and cleaning the enclosures are performed in a uniform manner by the same individuals to whom the animals are familiarly with.

**Table 1:** Jaguar details.

**Animal ID**	**Birth**	**Biome**	**Born in**	**Coat color**	**Reproductive History**
Merlin	Jan-2013	Cerrado	wild	melanistic	Blind; Mated with Jurema
Ferinha	Jul-2010	Amazonia	wild	non-melanistic	Several matting with no kittens
Guarani	Dec-2018	Pantanal	wild	non-melanistic	1 litter w/ 1 kitten
Aimará	Oct-2018	Pantanal	wild	non-melanistic	2 litters, 1 kitten each
Oxóssi	Mar-2020	Cerrado	captive	non-melanistic	1 female possible kittening

### Freezing media

The used media had been previously prepared by Reprodux Laboratórios Ltda (Itapira, SP, Brazil; 22°28'46.6"S 46°45'28.6"W). 10mL of Freezelip (Ref 026413; IMV Technologies) and 83,6 mL of Easy Buffer B (Ref 023862; IMV Technologies) were combined and mixed for 120 seconds to create the BASE solution. Prior to freezing, dimethylsulfoxide (Ref 023862; IMV Technologies), glycerol (Reprodux Laboratories), or methanol (Ref 026996; IMV Technologies) were added to semen diluted with BASE at a final concentration of 6.4%.

### Anesthesia

Males were deprived of water and fasted for 8 hours before procedures were conducted. A combination of a dissociative anesthetic from the cycloheximide group (ketamine 5mg/kg, im) and a sedative from the α-2 agonist group (medetomidine 0.1 mg/kg, im) was administered (Araujo et al., 2018, 2021b). Visual inspection was used to estimate animal weights, and individual doses were calculated based on these assumptions. For the administration of these medications, the animals were confined within the off-exhibit area of their respective enclosures, hence reducing the potential of chemical containment incidents following the delivery of the medication. Using a blowgun and pressurized darts, the anesthetic combination was delivered intramuscularly on the lateral side of the hind limbs. To safely perform the manipulation, 10 minutes were let to pass after the anesthetic injection, by which time the animals had lost their postural righting reflexes and no longer responded to auditory and tactile stimuli. To prevent aural and visual stimuli, the eyes and ears were covered, and the subject was transferred to a temperature-controlled procedure room (approximately 25 °C), where they were attached to a multiparametric monitor and positioned for semen collection. The animals were returned to their respective off-display areas after forty minutes of application of the anesthetic association - estimated action time of ketamine - and after semen collection. An α -2 agonist medication reverser (yohimbine 0.2 mg/kg, im) was administered to males to return them from chemical restraint in a shorter period of time. After being completely awake and able to move normally, the animals were returned to their enclosures, where they had access to water and were fed as usual.

### Infrared thermal imaging

Before beginning the andrological examination, infrared thermography (IRT) of the external rectal perineum, testes, and penis was captured using a FLIR One Pro LT camera (gen 3; Teledyne FLIR LLC) and FLIR One software (v. 4.0.1 for Android; FLIR Systems INC) as soon as the animal arrived in the procedure room. The chosen thermal color palette was the rainbow, in which warm colors represent the hottest areas of the image and cool colors represent the coldest; this palette is recommended for pinpointing surfaces in environments with minimal heat differences. To achieve more accuracy, manual calibration was performed by touching the Calibration icon in the upper right corner of the main program screen after turning on the camera and prior to snapping the image. The pictures were taken from a distance of approximately 60 cm. Three spot meters were utilized: the first (1) was placed in the outer region of the anal perineum, while the second (2) and third (3) were placed in the hottest regions of the right and left testicles, respectively. A multiparameter monitor (AM6100, Olieco China) was used to measure the esophagus temperature for comparison purposes.

To improve the optimal operating temperature of the post-collection sperm, a thermal picture was taken of the semen immediately after it exited the urethra. Two spot meters were established, the first at the outflow of the urethra and the second at the area of the probe with the highest temperature.

### Andrological evaluation

During the breeding soundness examination, the testicular biometry and consistency and penile spines were evaluated. Biometry was performed using a caliper, and testis consistency and penile spines (scale of 0 - no penile spines - to 3 - prominently visible) were evaluated subjectively. The testicular volume was measured using a caliper and applying the equation for an ellipsoid 4/3 × π × L/2 × B1/2 × B2/2, where L, B1, and B2 are the length and two breadths of the ellipsoid (Harcourt et al., 1995), then subsequently converted to grams (1.046) (Johnson et al., 1981). The relative testicular weight (RTW) was determined by dividing the total testes' weight by the body weight (Jorge-Neto et al., 2020c; Silva et al., 2022; Silvatti et al., 2020).

**Figure 1:**
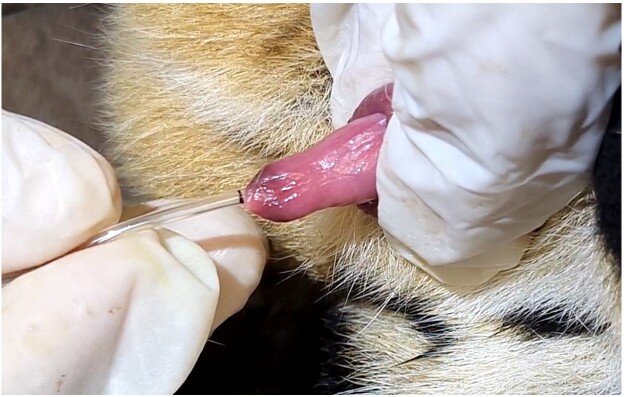
Urethral catheterization for jaguar semen collection. Photo: Pedro Nacib Jorge-Neto.

After 20 minutes of anesthetic induction with medetomidine, semen was extracted using the method described by Araujo et al. (2018), which entails retrieving sperm by urethral catheterization. With the exception of Aimará, who underwent two catheterizations separated by a 10-minute interval for semen recovery, the other animals were catheterized only once. Briefly, the penis was uncovered and cleansed with saline solution and gauze while the animal was being monitored. The urethra was then probed using a 4FR nasal oxygen catheter with a cut-off tip, which was inserted roughly 13 cm and positioned close to the prostate (Figure 1). Then, the semen was collected via capillarity or, if necessary, a 1 mL syringe was connected to the catheter, and gentle negative pressure was applied to aspirate the semen into the probe and syringe, after which it was placed in a 28 °C preheated microtube.

### Fresh semen assessment and freezing

The semen sample volume (µL) was measured using a variable volume pipette (Gilson Pipetman 10-100 µL), and the BASE solution at 28°C was added to the ejaculate in a 1:1 ratio (v/v) immediately after collection.

To assess seminal parameters in computer-assisted sperm analysis (CASA), 3µL of semen samples were diluted with the BASE solution to achieve a concentration of 20x10^6^ sperm/mL. The mixture was homogenized gently by shaking twice and then using a 2-200 µL pipette tip, 3µL of the sample was put into the chamber of a Leja slide (with the tip at 45°), and the external droplet was dry. Once the sample drift was stabilized, six fields in the center of the Leja slide (ref 025107, IMV Technologies) were analyzed using the CEROS II CASA system with the Animal Breeders II software (version 1.13.7, Hamilton Thorne) and using the specific setup for jaguars (Jorge-Neto et al., 2020a). The “Auto-First Frame” option was used to ensure the correct marking of sperms’ tails and heads.

Immediately following the assessment of the ejaculate concentration by CASA, the prediluted ejaculate (1:1, v/v) was completed with the BASE solution in the total volume at a concentration of 20M/mL, divided into three microtubes of equal volume and refrigerated at 4°C for two hours. After cooling, the cryoprotectants DMSO, GLY, and MET were added at a final concentration of 6.4% in the respective microtubes, and the doses were loaded into mini straws (IMV Technologies) and frozen in nitrogen vapor according to Araujo et al. (2020a) method. Frozen doses were stored in a liquid nitrogen tank until further analysis.

For phase contrast microscopy (Nikon Labophot Phase Contrast Microscope) assessment of sperm morphology, a sample of fresh sperm was fixed in a 4% formaldehyde solution in DMPBS (magnification ×1000). Per animal, approximately 100 cells (78 to 111) were evaluated. The sperm morphology was characterized according to the cattle-specific manual of the Brazilian College of Animal Reproduction.

### Assessment of thawed semen

Comparing temperatures of 37 °C for 30 seconds and 50 °C for 12 seconds, the doses from each treatment were thawed with a semen thawer (ref 450001, IMV Technologies Brasil). To prepare the sample for CASA analysis, each straw was thawed and completely emptied with a mandrel, diluted in a microtube with EasyBuffer B (1:1, v/v), and evaluated in the IVOS II CASA using the same software, setup, and method of sample preparation on a Leja slide as previously described.

### Statistical analysis

Using the Shapiro-Wilk test, data normality was assessed. ANOVA was used to test the effect of the semen cryoprotector and the thawing temperature on the sperm motility parameters since there was no indication of a violation in the normality of the sample. Using the R statistical program with the ggplot2, ggpubr, and dplyr packages, analyses were conducted (R Development Core Team, 2011).

## Results

**Figure 2:**
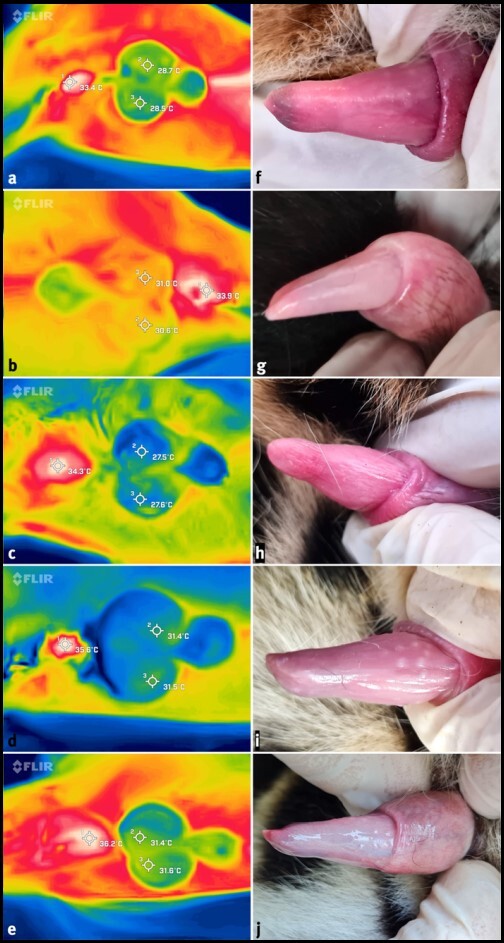
IRT of the testes (a to e) and penile spines (f to j) of the animals Merlin (a and f), Oxóssi (b and g), Guarani (c and h), Aimará (d and i), and Ferinha (e and j). Rainbow thermal color palette used. Photos: Pedro Nacib Jorge-Neto.

**Figure 3:**
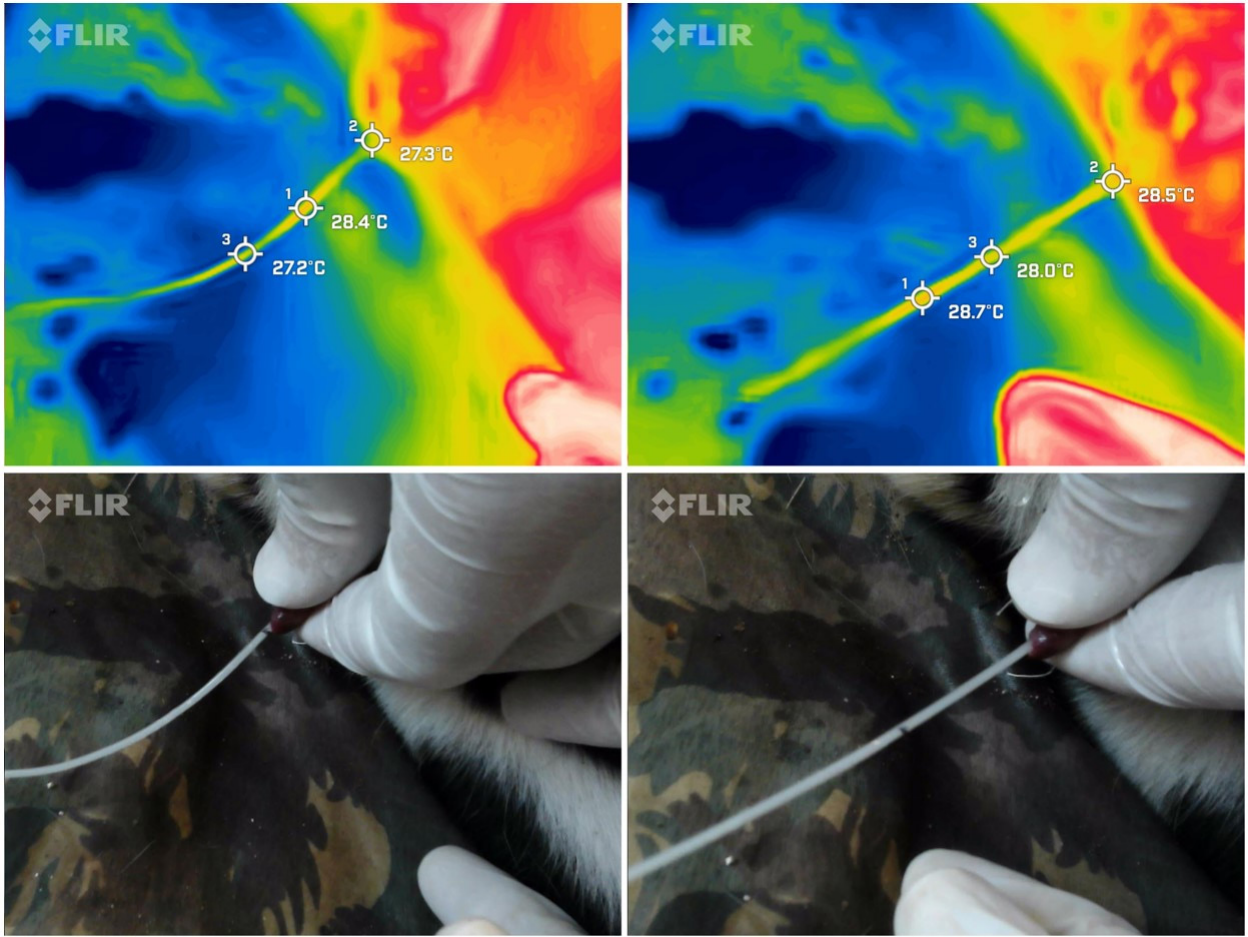
Post-collection seminal IRT. Rainbow thermal color palette used. Photos: Pedro Nacib Jorge-Neto.

The average esophageal temperature was 37.4 ± 1.2 °C, whereas the IRT of the exterior area of the anal sphincter was 34.7 ± 1.1 °C, resulting in a temperature differential of -2.3 ± 1.2 °C. The IRT average of both testicles was 30.0 ± 1.6 °C (Figure 2). Consequently, on average, the testes' superficial temperature was 4.6 ± 1.2 °C cooler than the anal sphincter. The temperature of the semen immediately after exiting the urethra was determined to be between 27.3 and 28.7 °C (Figure 3).

Except for Ferinha, who had a hard testicular consistency, the others exhibited a typical fibroelastic consistency. Oxóssi's testicular biometry could not be performed due to a lack of time to return him to his enclosure prior to his awakening from anesthesia. The volumes of the right and left testicles were 8.8 ± 3.1 and 9.6 ± 1.8 cm^3^, the total volume of the testes was 18.4 ± 4.8 cm^3^, and the relative testes weight was 0.27 ± 0.05 cm^3^/kg (Table 2). Two older males had few penile spines, whereas two of the three younger males had none, and one had medium-developed spines (Table 3).

**Table 2:** Jaguar testicular biometry results.

**Animal ID**	**Age (month)**	**Weight (kg)**	**RTV (cm^3^)**	**LTV (cm^3^)**	**MTV (cm^3^)**	**TTV (cm^3^)**	**RTW (cm^3^/kg)**
Merlin	110	70	13.25	11.23	12.240	24.480	0.35
Ferinha	140	49	5.40	7.62	6.511	13.022	0.27
Guarani	39	82	8.93	10.64	9.786	19.573	0.24
Aimará	41	63	5.52	7.23	6.375	12.749	0.20
Oxóssi	24	70	-	-	-	-	-
MEAN	70.8	68.8	6.6	7.3	7.0	14.0	0.21
SD	45.6	10.8	4.4	4.0	4.1	8.2	0.12

RTV: right testicular volume; LTV: left testicular volume; MTV: mean testicle volume; TTV: total testicular volume; RTW: relative testicle weight; SD: standard deviation.

**Table 3:** Spines, temperature and IRT.

**Animal ID**	**Spines**	**ET**	**AS-IRT**	**AT-IRT**	**≠ET-AS**	**≠AS-AT**
Merlin	few	36.6 °C	33.4 °C	28.6 °C	-3.2 °C	-4.9 °C
Ferinha	few	39.6 °C	36.2 °C	31.5 °C	-3.4 °C	-4.6 °C
Guarani	none	36.3 °C	34.3 °C	27.6 °C	-2.0 °C	-6.7 °C
Aimará	medium	37.6 °C	35.6 °C	31.5 °C	-2.0 °C	-4.1 °C
Oxóssi	none	36.9 °C	33.9 °C	30.8 °C	-3.0 °C	-2.9 °C
MEAN		37.4 °C	34.7 °C	30.0 °C	-2.7 °C	-4.6 °C
SD		1.2 °C	1.1 °C	1.6 °C	0.6 °C	1.2 °C

ET: esophageal temperature; AS-IRT: anal sphincter infrared thermography; AT-IRT: average testes infrared thermography; ≠ET-AS: difference between esophageal temperature and anal sphincter infrared thermography; ≠AS-AT: difference between anal sphincter and average testes infrared thermography; SD: standard deviation.

The urethral catheterization approach for collecting semen was effective for all males. On average, 25 minutes after the administration of medetomidine, the first catheterization was performed. With the exception of Aimará, from whom two distinct ejaculates could be obtained, only one sample was collected from each male. The average amount of semen collected was 117.8 ± 54.7 µL, with a total motility of 55.3% ± 22.6% and a progressive motility of 36.3% ± 18%. The concentration of sperm was 2,344 ± 1,613 x 10^6^ per milliliter (Table 4).

**Table 4:** Results of jaguar fresh seminal parameters.

**Animal ID**	**Volume (µL)**	**Total Motility (%)**	**Progressive Motility (%)**	**Sperm/mL (x10^6^)**	**Total Spz (x10^6^)**
Merlin	54	21.4	10.3	2893	156
Ferinha	180	27.1	17.2	51	9
Guarani	196	63.9	39.0	1521	298
Aimará CT 1	122	75.3	57.1	1951	238
Aimará CT 2	60	65.3	35.6	5380	323
Oxóssi	95	78.9	58.0	2270	216
MEAN	117.8	55.3	36.2	2344	207
SD	54.7	22.6	18.0	1613	104

Comparing the types of cryoprotectants (Table 5), DMSO and GLY did not differ on the motility parameters in the CASA analysis (P= 0.468 however, when comparing the sperm motility using the cryoprotectant MET with DMSO and GLY a significant difference was observed (P<0.05). Visual subjective analysis reveals that sperm treated with DMSO have greater vigor.

**Table 5:** Effect of the cryoprotector on total and progressive motility after thawing.

**Cryoprotector**	**n**	**Total Motility (%)**	**Progressive Motility (%)**
DMSO	13	5.28 ± 2.51^a^	1.22 ± 0.89 ^c^
GLY	12	4.29 ± 2.49 ^a^	1.26 ± 1.14 ^c^
MET	12	0.51 ± 0.62 ^b^	0.02 ± 0.06 ^d^

Different superscript letters indicate statistical differences (p < 0.05) in sperm quality parameters between cryoprotectors.

Comparing the semen thawing temperatures of 37 °C for 30 seconds and 50 °C for 12 seconds with the same diluent, there was no difference (Table 6).

**Table 6:** Total and progressive post-thawing motility by thawing temperature.

**Cryoprotector**	**Temperature °C**	**N**	**Total Motility (%)**	**Progressive Motility (%)**
DMSO	37	5	5.1 ± 3.14 ^a^	1.42 ± 1.28 ^c^
DMSO	50	8	5.4 ± 2.27 ^a^	1.1 ± 0.62 ^c^
GLY	37	4	3.92 ± 3.39 ^a^	0.8 ± 1.23 ^c^
GLY	50	8	4.47 ± 2.17 ^a^	1.49 ± 1.1 ^c^
MET	37	5	0.56 ± 0.56 ^b^	0.04 + ± 0.09 ^d^
MET	50	7	0.47 ± 0.7 ^b^	0 ± 0 ^d^

Different superscript letters indicate statistical differences (p < 0.05) in sperm quality parameters between treatments.

All tested animals had a high proportion of spermatozoa with morphological changes (Table 7), with the proportion of normal spermatozoa ranging from 8% to 45%. The morphological change that occurred most frequently was pseudo droplet, followed by simple bent tail and proximal cytoplasmic droplets (Table 7).

**Table 7:** Types of morphological changes in fresh jaguar semen.

**Percentage (%)**	**Merlin**	**Ferinha**	**Guarani**	**Aimará EJ1**	**Aimará EJ2**	**Oxóssi**
Absent head	1	1	-	1	-	-
Knobbed acrosome	4	-	3	2	5	-
Abnormal form head	1	9	11	7	2	-
Tapered heads	-	2	-	-		-
Normal destached head	-	2	-	-		-
Microcephaly	-	2	-	-		-
Abnormal small head	2	-	-	2	1	-
Vacuole	-	1	-	-		1
Aplastic midpiece defect	1	2	-	-		-
Corkscrew	-	-	-	-	1	1
Aplasia of the mitochondrial sheath	-	-	3	-		-
Swelling midpiece	3	2	6	1		7
Espessamento	-	2	-	-		-
Fracture midpiece	-	6	-	1		-
Pseudo droplet	56	6	37	55	47	44
Oblique	-	1	-	-		-
Simple bent tail	6	10	4	13	5	9
Simple coiled tail	-	3	1	1		-
Bent principal piece	1	1	2	3	2	3
Coiled principal piece	1	2	2	4	3	4
DAG defect	1	-	-	-	1	-
Underdeveloped	1	-	-	-	1	-
Proximal cytoplasmic droplets	19	9	7	4	4	3
Total morphological defects	97	59	111	101	92	84
Sperm with defects	92	55	89	88	83	84
Normal sperm	8	45	11	12	17	16

## Discussion

The first hurdle for the development of reproductive biotechnologies is the collection and analysis of sperm (Barnabe et al., 2002). Collecting representative quantities of sperm from wild felines is challenging due to the inapplicability of domestic animal procedures.

In this study, IRT was utilized to determine the specific thermal profile of the jaguar testis in order to adopt the theoretically optimal temperature that is closer to the epididymal temperature when working with collected sperm. Consequently, we confirmed that the difference between the internal and anal sphincter temperatures is -2,3 ± 1,2 °C. In addition, the testes have a surface temperature that is 4.6 ± 1.2 °C lower than the anal sphincter. The measurement of the sperm's temperature immediately after exiting the urethra (between 27.3 and 28.7 °C) enabled us to identify 28 °C as the optimal temperature for handling the collected sperm. As far as we know, this is the first study to investigate IRT in the *Panthera* genus.

Males in this study had few or no penile spicules, but all displayed entire copulatory behavior (Jorge-Neto et al., 2018), indicating sexual maturity. Aimará and Oxóssi had the best sperm quality, with total motility of 75.3% and 78.9% and progressive motility of 57.1% and 58.0%, respectively. Surprisingly, they were the males with the maximum number of sperm collected and lack of penile spines, as well as the parents of both offspring at the moment of the procedure. The other animals possessed none or few spines. Araújo et al. (2021a) report observing the lack or scarcity of penile spines in adult jaguars in both *ex situ* and *in situ* environments, despite the production of high-quality semen. Consequently, the absence or scarcity of penile spines does not render reproduction unfeasible, especially given that ovulation in the jaguar can be induced by sensory stimuli (Jorge-Neto et al., 2020b) and by the multiple copulations during the female estrus period (Jorge-Neto et al., 2018). Photographs of the penile spines of *Panthera* species have rarely been published. This study's males have penile spines comparable to those reported by Silva et al. (2019a) in jaguars. In addition, they had traits with the penile spines of other species from the same genus, including the Javan leopard *P. pardus melas* (Mulia et al., 2021), the Arabian leopard *P. pardus nimr* (Baqir et al., 2015), and the African lion *P. leo* (Schepper, 2016), but they were less numerous than other neotropical cat species of the genus Leopardus (Araujo et al., 2013; Machado et al., 2020; Morais et al., 2002).

Araujo et al. (2018) established in jaguars the pharmacological semen collection by combining medetomidine and ketamine. In addition to allowing excellent sperm recovery, this anesthetic protocol has also been selected for general treatments in the species, both in captive and free-living animals. The possibility of reversing anesthesia with yohimbine or atipamezole has led to its use in females (Luczinski et al., 2020) for laparoscopic ovum pick-up, which has proven to be a highly effective and safe protocol.

Currently, pharmacological semen collection by urethral catheterization is the gold-standard method for obtaining semen in wild felines (Araujo et al., 2018, 2020b; Jeong et al., 2018; Jorge et al., 2019; Kheirkhah et al., 2017; Lueders et al., 2012), other wild species (Araujo et al., 2023; Curry and Roth, 2016; Jeong et al., 2019; Silinski et al., 2002; Silva et al., 2022) and in domestic species (Cavalero et al., 2019; Madrigal-Valverde et al., 2021; Silva et al., 2021), and it allowed efficient semen recovery from the five male jaguars in this study. The first step in the development of assisted reproduction technology for a species is the effective collection of sperm, which aims to permit, in the future, the growth of genetic variety across *in situ* and *ex situ* populations, embracing the concept of One Conservation (Pizzutto et al., 2021). Reproductive biotechnologies and fertility preservation are essential tools for the conservation and maintenance of endangered species (Comizzoli, 2017). Therefore, the successful cryopreservation of jaguar sperm enables the establishment of a biobank containing material with a greater likelihood of future application.

To date, only glycerol has been utilized in jaguars as sperm cryoprotector, as well as other carnivores, in quantities ranging from 2% to 10% (Silva et al., 2019b). However, DMSO proved more efficacious in maned wolves' *Chrysocyon brachyurus* semen than glycerol (Johnson et al., 2014) but declines sperm total and progressive motility in red wolf *Canis rufus* (Franklin et al., 2018). Nevertheless, our findings confirm those of a different *Panthera* genus species, the African lion, in which there was no significant difference between DMSO and GLY (Stander, 2004). Different commercial sperm extenders were tested on jaguars by the Reprocon Institute (unpublished data) in the past, with OptiXcell standing out. It is an egg yolk-like media but with synthetic LDL, allowing for an excellent evaluation of the sperm in the CASA system due to the absence of yolk components that produce artifacts during the assessment. Therefore, we attempted to formulate a media similar to that of OptiXcell, but with a different cryoprotectant. Our data reveal that, although there is no statistical significance between GLY and DMSO, the last results in better visual sperm vigor. Our results concur with Buranaamnuay’s (Buranaamnuay, 2020), who reported that methanol is a less effective cryoprotectant than DMSO or glycerol for domestic cats. Therefore, further studies should investigate the effect of other cryoprotectants in jaguars' and other felines' semen, in addition to different DMSO concentrations.

The typical thawing temperature for jaguars is 37 °C. Knowing the low motility of this species' sperm after thawing, we attempted to improve the quality of the thawed sperm by increasing the thawing temperature. In several species, an increase in thawing temperature enhanced the viability and motility of the sperm (Cochran et al., 1984; Dhami et al., 1992; Iaffaldano et al., 2016). Nonetheless, our results did not indicate an improvement in the outcomes. The temperature of 50°C was determined based on the maximum temperature reached by the semen thawer, with the stated goal of maintaining the standard temperature throughout all procedures. The quality of the thawed dose should also be evaluated at temperatures up to 90 °C for 5 seconds in future research.

Few studies have assessed frozen-thawed jaguar spermatozoa, with three grading sperm subjectively by eyes (Paz et al., 2007, 2000; Swanson et al., 1996) and two using CASA systems (Araujo et al., 2020a; Silva et al., 2020). Traditional evaluation of semen under a microscope by eyes is vulnerable to the subjective discriminating levels of technicians, which contributes to the substantial variation across studies in conventional sperm analysis. Broekhuijse et al. (2011) state that conventional semen evaluations were subjective and incomparable to data provided by CASA when standardized, in addition to being less accurate. The CASA system can rapidly evaluate a large number of spermatozoa, delivering more accurate and quantitative data on sperm cell dynamics and enhancing the reliability of the analyses. On the other hand, CASA requires a species-specific setup, sample preparation processes, and the type of slide utilized for an accurate analysis. Total motility can be overestimated when a sample with a high sperm concentration is assessed, as faster-moving cells can collide with static spermatozoa that seem motile.

Consequently, the analysis with a concentration near 20x10^6^ sperms per milliliter avoids this incorrect assessment. The influence of axis also has a negative impact on sperm motility assessment when slides and coverslips are used in front of high-precision disposable counting chambers. Using slides with coverslips, Rouillon et al. (2020) discovered that the total motility was 61% in the center of the slide and 35% on the margins. With disposable high-precision counting chambers, this rheotaxis effect does not occur. In this investigation, the quality of thawed semen was significantly inferior to that of other studies. For the reasons stated above, it is not possible to compare our results to those of research that subjectively assessed sperm motility by eyes. When we compare our findings to those of the two studies that employed CASA, we find that progressive motility is comparable, but overall motility differs substantially. We believe that this difference is due to the type of slide and different sample preparation methods, which were not specified in those publications, as well as the nonexistence of a species-specific setup and the use of older versions of the CASA system, with software that lacked additional options for creating species-specific setups. In contrast, in this study, in addition to a setup designed exclusively for jaguars, the equipment and the software version used are the most recent versions available to date, and operating procedures were precisely detailed and explained, including the use of high-precision disposable counting chambers.

Although teratospermia is uncommon among jaguars (Araujo et al., 2018; Gonzalez et al., 2017; Morato et al., 2001), we observed it in four out of five males. Surprisingly, the normospermic animal (Ferinha) is the only one confirmed to be infertile, as none of the several coupling efforts resulted in females becoming pregnant or in early embryonic death when pregnancy was not yet detectable. All other males, however, revealed morphological defects in their sperm at a rate of >80%, and two of them (Guarani and Aimará), upon copulating with the same females as Ferinha, had offspring at the time of semen collection. Ferinha was mated with Jurema (98 mo), Cabocla (110 mo), and Jaci (146 mo) multiple times over the years and during their numerous estrous cycles. There was never a case of a female becoming pregnant, as the estrus was repeated every 30 days on average. Cabocla and Jaci were coupled with the Aimará after unsuccessful copulations with Ferinha, and both fell pregnant in the first estrus. Jurema was then matched with Merlin, and the two mated during her first heat. Jurema had no signs of heat after 30 days, but she copulated with Merlin again after approximately 60 days. Jurema has not undergone estrus in the past 50 days, but a pregnancy diagnosis has not been made so far. Oxóssi was mated and copulated with Yasmin (134 mo) during her first heat, and she did not return to heat. She had breast enlargement and had prepared a nest for kittening, but no live kittens were found. It is known that felines can commit infanticide (Ruiz-Olmo et al., 2018; Veronesi and Fusi, 2022) or eat stillborn (Laurenson, 1994; Sankhala, 1967). Guarani and Amanaci formed the sole ex situ couple of jaguars from the Pantanal biome in the world. They copulated during their first heat, and she gave birth to a pup that should be released into the wild. As both were born free-living, their reproductive performance was the example of success reported by Luczinski et al. (2023) of the One Conservation approach.

We consider sperm defects to be morphological changes that interfere with male fertility. The jaguar's reproductive behavior is not conducive to the production of high-quality sperm, as males ensure copulation (and progeny) with the female by retaining her inside their territory. Additionally, multiple copulations increase the number of sperm that are deposited in the female reproductive tract at the moment of ovulation. Our data clearly demonstrate that sperm morphologic changes have a limited impact on jaguar fertility. Therefore, we refute the applicability of bovine semen morphological classifications to felids and propose the development of a specific classification.

## Conclusion

The total and progressive motility measured by CASA did not differ between the cryoprotectants DMSO and glycerol, but they were superior to methanol. The assessment of two different thawing temperatures (37 °C and 50 °C) revealed no differences between each other, regardless of the cryoprotectant employed.

The thawed sperm exhibited low levels of total and progressive motility. Males with a high level of sperm morphological alterations were found to be fertile, but the sole male with normospermia was found to be infertile. Thus, we contest the applicability of the commonly employed morphological classification created for bovines to felids species. The most prevalent morphological abnormalities were pseudodrops, followed by bent tails and proximal cytoplasmic droplets.
